# The Asiatic Cholera

**Published:** 1846-06

**Authors:** 


*The Asiatic Cholera.*—In a late number we stated that the Asiatic cholera had spread through several provinces of Persia, and had given rise to great mortality in some of the principal towns. It is reported to have extended from Bokhara across the Persian frontier to Herat and Meshid, thence south of the Caspian to Teheran, and still further south to Ispahan. Recent accounts from Odessa, state that it has crossed the Russian boundary, and has appeared at Tiflis, taking a course northward between the Caspian and Black seas; while according to the latest intelligence from Riga, it has broken out at Orenburg in the Uralian mining district, crossed the Volga, and appeared on the European side at Kasan, about 1200 miles from St. Petersburg. If these accounts are to be trusted, the disease has taken a somewhat irregular course, in a direction west by north; and it does not appear to have followed the banks of great rivers as in the former irruption of 1828-30. The disease which reached England in 1831, prevailed in Persia for seven years from 1823 to 1830. It appeared at Orenburg for the first time in 1823; and was confined to this quarter for a period of five years. It reappeared at Orenburg in 1829, and its prevalence and fatality in this province were so great, that upwards of one-tenth of the inhabitants were seized with it, and one-fourth of those who were attacked, died. It reached St. Petersburg in July, 1831, and England on the 26th October of that year. At Tiflis, where it is again reported to have broken out, the mortality from the former epidemic was so great, that three-fourths of those who were attacked, perished. We shall take care to report any further information which may reach us respecting the progress of the malady.—*Ibid.*

